# A Tooth at Birth

**Published:** 1883-08

**Authors:** 


### A TOOTH AT BIRTH.

Dr. Fordyce Grinnell, of Pine Ridge Agency, Dakota, reports the following case in the *Med. News,* March 31, 1883:

I drew a tooth for an infant seventeen days old. The child was a female, one-quarter Sioux. When it came into the world it had this tooth, a lower central incisor, developed as now presented. The crown and body of the tooth seemed fully developed, and as large as the usual milk tooth. The attachment to the gum, instead of a root, appeared to be a mere fleshy connection, and hence not very solidly implanted. Moreover, it gave the child great uneasiness in nursing. The tongue in moving over the sharp surface presented by the tooth in every attempt at drawing milk from the breast, became abraded, and finally ulcerated. As soon as the tooth was extracted the child took the breast with avidity. The pain caused by nursing had undoubtedly interfered with the child getting a sufficient amount of nourishment.—*Louisville Med. News.*

